# Identification of Key Genes and Long Noncoding RNA-Associated Competing Endogenous RNA (ceRNA) Networks in Early-Onset Preeclampsia

**DOI:** 10.1155/2020/1673486

**Published:** 2020-06-05

**Authors:** Zhan Zhang, Ping Wang, Linlin Zhang, Chenxi Huang, Junjun Gao, Yingying Li, Bo Yang

**Affiliations:** ^1^Department of Clinical Laboratory, The Third Affiliated Hospital of Zhengzhou University, Zhengzhou, 450052 Henan Province, China; ^2^Department of Medical Research Center, The Third Affiliated Hospital of Zhengzhou University, Zhengzhou, 450052 Henan Province, China

## Abstract

**Background:**

Preeclampsia (PE) is a pregnancy-specific hypertension syndrome and is one of the leading causes of maternal and perinatal morbidity and mortality. Long noncoding RNAs (lncRNAs) have been reported to be abnormally expressed in many diseases, including preeclampsia. The present study is aimed at identifying the key genes and lncRNA-associated competing endogenous RNA (ceRNA) networks in early-onset preeclampsia (EOPE).

**Methods:**

We investigated expression profiles of differentially expressed lncRNAs (DElncRNAs) and genes (DEGs) in placental tissues of EOPE and healthy controls with Human LncRNA Array v4. The potential functions of DEGs and DElncRNAs were predicted using the clusterProfiler package. The lncRNA-mRNA coexpression network was constructed via Pearson's correlation coefficient. The protein-protein interaction (PPI) network of DEGs was constructed, and the hub genes were obtained using the STRING database and Cytoscape. The ceRNA networks were constructed based on miRWalk and LncBase v2. qRT-PCR was performed to confirm the expression of lncRNA MIR193BHG, PROX1-AS1, and GATA3-AS1. ROC curves were performed to assess the clinical value of lncRNA MIR193BHG, PROX1-AS1, and GATA3-AS1 in the diagnosis of EOPE.

**Results:**

We found 6 hub genes (SPP1, CCR2, KIT, ENG, ACKR1, and FLT1) altered in placental tissues of EOPE and constructed a ceRNA network, including 21 lncRNAs, 3 mRNAs, and 69 miRNAs. The expression of lncRNA MIR193BHG and GATA3-AS1 were elevated and showed good clinical values for diagnosing EOPE.

**Conclusion:**

This study provides novel insights into the lncRNA-related ceRNA network in EOPE and identified two lncRNAs as potential prognostic biomarkers in EOPE.

## 1. Introduction

Preeclampsia (PE) is a pregnancy-specific complication associated with new-onset hypertension, affecting an estimated 4-5% of pregnancies worldwide [1]. It often presents with proteinuria or other end-organ damages in the mother after 20 weeks of gestation [2]. PE is one of the leading causes for pregnancy-associated morbidity and mortality as it may cause prematurity of the fetus and long-term cardiovascular disease (CVD) in the mother [3]. PE can be classified into early-onset PE (EOPE; <34 weeks) and late-onset PE (LOPE; ≥34 weeks). Many studies have shown that poor placental development is associated with EOPE, which is a more severe form and has more adverse outcomes [4]. However, the exact molecular mechanisms of EOPE have not been completely elucidated. Thus, exploring key genes for EOPE is essential to disclosing the underlying molecular mechanisms and investigating possible diagnostic and therapeutic targets.

Long noncoding RNAs (lncRNAs), a subclass of ncRNAs with a length of more than 200 nucleotides, play essential roles in regulating various biological functions [5]. The alteration and dysregulation of several lncRNAs have been found to be associated with PE, such as SPRY4-IT1, RP11-465L10.10, MALAT1, H19, and HELLP [6, 7]. However, researches on the function and mechanism of lncRNAs in EOPE are scarce, and the exact contributions of lncRNAs to EOPE remain largely unknown. One of the well-defined functions of lncRNAs is that lncRNAs may act as competing endogenous RNAs (ceRNAs) to sponge microRNAs (miRNAs), thus affecting the regulatory function of miRNAs and imposing posttranscriptional regulation of gene expression [8]. However, specific lncRNAs and their related ceRNA network are scarcely investigated in EOPE.

In this study, we aimed to identify the key genes and construct pivotal lncRNA-related ceRNA networks in EOPE. We analyzed lncRNA and mRNA expression profiles in the placenta of EOPE and healthy controls based on the microarray technology. Biological function and interactions between differentially expressed lncRNAs (DElncRNAs) and genes (DEGs) were studied using comprehensive bioinformatic analysis. By analyzing the PPI network, we screened 6 hub genes. Based on coexpression analysis and online prediction, we constructed a lncRNA-miRNA-mRNA network (ceRNA network) including 3 hub genes, 21 lncRNAs, and 69 miRNAs. Furthermore, we confirmed that lncRNA MIR193BHG and lncRNA GATA3-AS1 from the ceRNA network were overexpressed and had potential values to be biomarkers for diagnosing EOPE.

## 2. Materials and Methods

### 2.1. Tissue Sample Collection

Placental tissue samples were collected during caesarean delivery from EOPE patients (*n* = 30) and healthy controls (*n* = 30) at the Third Affiliated Hospital of Zhengzhou University from May 2018 to July 2019. EOPE was defined as new-onset hypertension (systolic blood pressure ≥ 140 mmHg and/or diastolic blood pressure ≥ 90 mmHg) with or without proteinuria (≥300 mg/24 h) which occurs after 20 weeks and prior to 34 weeks of gestation [9]. The exclusion criteria included multiple gestations, gestational diabetes mellitus, chronic hypertension, thyroid dysfunctions, and kidney disease. Placental tissues were obtained immediately after caesarean delivery. After washing with cold PBS, these tissues were stored in RNAstore (CWBio, China) at -80°C. This study was approved by the Ethics Committee of the Third Affiliated Hospital of Zhengzhou University. All samples were collected with the patients' informed consents and written approvals.

### 2.2. RNA Extraction and Microarray Experiments

CapitalBio Technology Human LncRNA Array v4 (4 × 180 K) was used in this experiment. Total RNA was extracted from placental tissues using the TRIzol Reagent (CWBio, China). RNA purity and concentration were spectrophotometrically determined from OD260/280 readings using a NanoDrop ND-1000. RNA integrity was assessed by capillary electrophoresis using the Bioanalyzer 2100 and the RNA 6000 Nano LabChip Kit (Agilent Technologies Inc., Santa Clara, CA, USA). RNA amplification, labeling, and hybridization were conducted according to the manufacturer's instructions. Briefly, double-stranded complementary DNA was transcribed from total RNA, then synthesized into complementary RNA and labeled with a fluorescent dye (Cy3-dCTP). The labeled complementary RNAs were hybridized onto the microarray. After washing, the arrays were scanned by the Agilent Scanner G2505C (Agilent Technologies Inc.).

### 2.3. Microarray Analysis

Microarray data were analyzed for data summarization, normalization, and quality control using the GeneSpring software, V13.0 (Agilent Technologies Inc., Santa Clara, USA). lncRNAs or mRNAs with statistical significance between EOPE and the control group were identified with a threshold of a ∣fold change | >2.0 and *P* < 0.05.

### 2.4. Gene Ontology (GO) and Kyoto Encyclopedia of Genes and Genomes (KEGG) Pathway Analyses

The clusterProfiler (http://bioconductor.org/packages/release/bioc/html/clusterProfile-r.html) is an ontology-based R package that not only automates the process of biological-term classification and enrichment analysis of gene clusters but also provides a visualization module for displaying analysis results [10]. In the present study, the clusterProfiler package was used to identify and visualize the top GO terms and KEGG pathways enriched by DEGs and DElncRNAs.

### 2.5. Construction of the lncRNA-mRNA Coexpression Network

The Pearson correlation coefficient [11] of each DElncRNA and DEG was calculated and the correlation test was performed to screen the lncRNA-mRNA pairs using ∣*r* | >0.9 (*P* < 0.05). These pairs were considered to have a coexpression relation, and the coexpression network was visualized with Cytoscape V3.7 (http://www.cytoscape.org/) [12].

### 2.6. Construction of the Protein-Protein Interaction (PPI) Network of DEGs

The PPI network of DEGs was constructed using the Search Tool for the Retrieval of Interacting Genes (STRING) database (http://www.string-db.org) [13]. The minimum required interaction score was set as 0.4. Cytoscape software was utilized to display the PPI network. We screened the top 6 genes with the highest degree of connection to the others as hub genes using cytoHubba from Cytoscape.

### 2.7. Construction of the ceRNA Network

The mRNAs in the ceRNA network were the hub genes screened in the PPI network. The miRNAs that targeted the mRNAs were retrieved from miRWalk (http://mirwalk.umm.uni-heidelberg.de/) [14]. The lncRNA-miRNA interactions were predicted with the LncBase Predicted v.2 (http://carolina.imis.athena-innovation.gr/diana_tools/web/index.php?r=lncbasev2%2Findex-predicted) [15]. By integrating the lncRNA-mRNA, lncRNA-miRNA, and mRNA-miRNA pairs, the lncRNA-miRNA-mRNA ceRNA network was constructed and visualized using Cytoscape.

### 2.8. Quantitative Real-Time Polymerase Chain Reaction (qRT-PCR)

Total RNA was extracted using the aforementioned method, and then 1 *μ*g RNA was reverse transcribed into cDNA using the ReverTra Ace qPCR RT Kit (Toyobo, Japan) according to the manufacturers' instructions. Specific primers were used to synthesize the cDNA of miRNAs. QRT-PCR was performed with the SYBR Green Realtime PCR Master Mix (Toyobo, Japan) in 20 *μ*L reactions. All reactions were performed on a StepOnePlus™ Real-Time PCR System under the following conditions: 95°C for 60 seconds, 40 cycles of 95°C for 15 seconds, 60°C for 15 seconds, and 72°C for 45 seconds. The results were normalized to GAPDH. The relative changes of all the target genes were calculated using the 2^−ΔΔCT^ method. The primers for MIR193BHG (TCONS_00024336) were as follows: forward, AGGGGCTGATGAATTGAGGG; reverse, TCAATGGCAGCAGGAGGTTA. The primers for PROX1-AS1 (ENST00000598091.1) were as follows: forward, ACAGGGAGCAGGTGACAGAGAC; reverse, TGAGGCAGACACGGGTCAGTC. The primers for GATA3-AS1 (TCONS_00017684) were as follows: forward, AAGTTGAGCGGGGTATGT; reverse, TTTCTGGCCTTTGGTGTC. The primers for GAPDH were as follows: forward, AGAACGGGAAGCTTGTCATC; reverse, CATCGCCCCACTTGATTTTG.

### 2.9. Statistical Analysis

Data were presented as mean ± SD. SPSS 21.0 and GraphPad Prism 6.0 were used for statistical analysis. Student's *t*-test (two tailed) was conducted to compare the difference between the two groups. *P* < 0.05 was considered statistically significant.

## 3. Results

### 3.1. Data Expression

**Figure 1:**
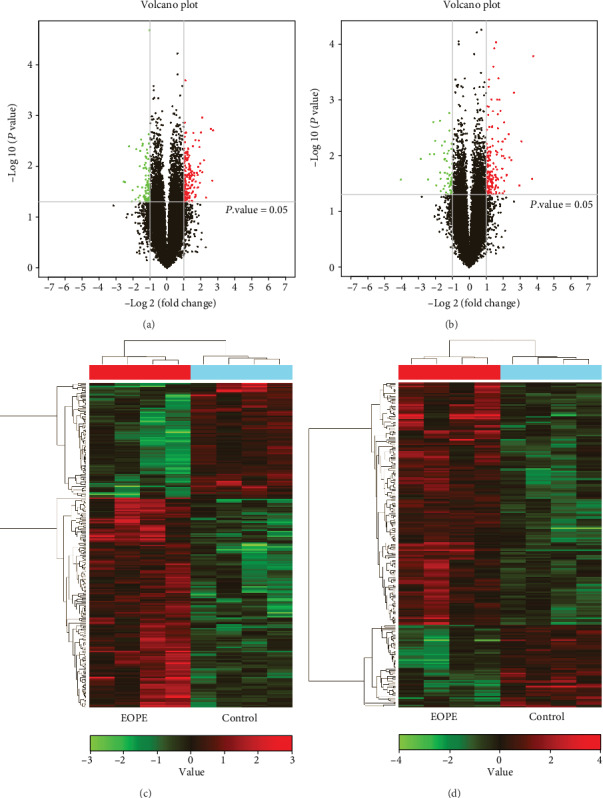
Identification of DElncRNAs and DEGs. Volcano plot of (a) DElncRNAs and (b) DEGs in placental tissues of EOPE and the control group (∣fold change | >2; *P* < 0.05). Red plots represent significantly upregulated lncRNAs and mRNAs. Green plots represent significantly downregulated lncRNAs and mRNAs. Heat maps of (c) DElncRNAs and (d) DEGs in placental tissues of EOPE and the control group (∣fold change | >2; *P* < 0.05). Red represents upregulated lncRNAs and mRNAs. Green represents downregulated lncRNAs and mRNAs. lncRNA: long noncoding RNA; mRNA: messenger RNA; EOPE: early-onset preeclampsia; DElncRNA: differentially expressed lncRNA; DEG: differentially expressed gene.

Microarray analysis was performed to determine lncRNA and mRNA levels in EOPE samples and controls. The clinical characteristics of these patients are presented in Table 1. DElncRNAs or DEGs were filtered with a threshold of a ∣fold change | >2.0 and a *P* value < 0.05 (Figures 1(a) and 1(b)). As a result, we identified 246 DElncRNAs, including 159 upregulated lncRNAs and 87 downregulated lncRNAs (Figure 1(c)), and 224 DEGs, including 167 upregulated mRNAs and 57 downregulated mRNAs (Figure 1(d)).

### 3.2. Functional Enrichment Analysis

**Figure 2:**
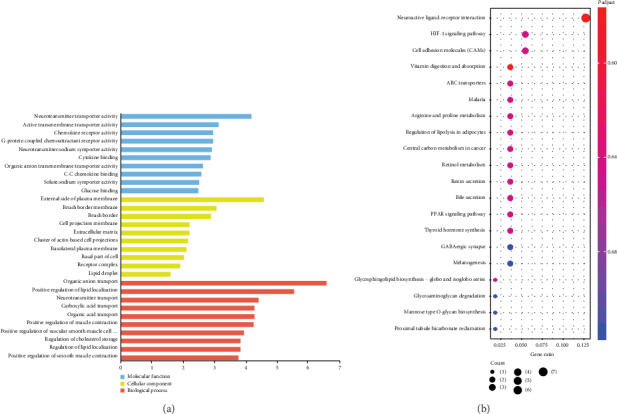
GO term enrichment and KEGG pathway analyses of DEGs. (a) GO annotations of DEGs with top 10 enrichment scores. (b) Top 10 KEGG pathway enrichment of DEGs. GO: gene ontology; KEGG: Kyoto Encyclopedia of Genes and Genomes; DEG: differentially expressed gene; EOPE: early-onset preeclampsia.

GO analysis covers three domains: cellular component, biological process, and molecular function. For further insight into the major biological function of dysregulated genes in EOPE, GO and KEGG analyses were performed. GO analysis showed the DEGs were mainly involved in organic anion transport, positive regulation of lipid localization, and neurotransmitter transport (Figure 2(a)). KEGG pathway analysis indicated the DEGs were intensively associated with neuroactive ligand-receptor interaction, HIF-1 signaling pathway, cell adhesion molecules (CAMs), and vitamin digestion and absorption (Figure 2(b)).

### 3.3. Construction of the lncRNA-mRNA Coexpression Network and Functional Prediction of DElncRNAs

**Figure 3:**
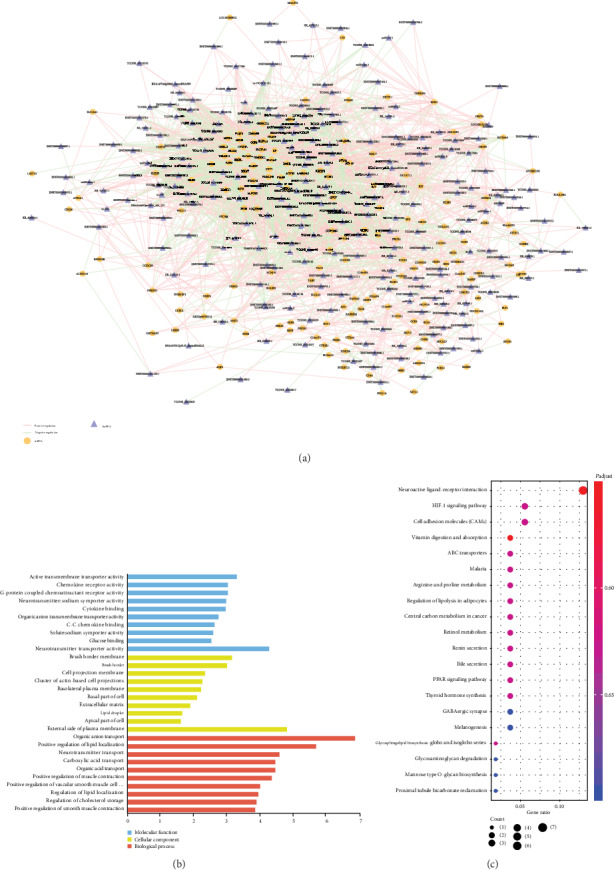
lncRNA-mRNA coexpression network and functional prediction of DElncRNAs. (a) lncRNA-mRNA coexpression network. (b) GO annotations of DElncRNAs with top 10 enrichment scores. (c) Top 10 KEGG pathway enrichment of DElncRNAs. GO: gene ontology; KEGG: Kyoto Encyclopedia of Genes and Genomes; lncRNA: long noncoding RNA; mRNA: messenger RNA; DElncRNA: differentially expressed lncRNA.

Based on the Pearson correlation test, a lncRNA-mRNA coexpression network was constructed and visualized by Cytoscape (Figure 3(a)). GO and KEGG analyses of these mRNAs were applied to predict the function of DElncRNAs. The results indicated that those DElncRNAs mainly regulated organic anion transport and neurotransmitter transport (Figure 3(b)). KEGG pathway analysis showed that DElncRNAs were enriched in neuroactive ligand-receptor interaction, HIF-1 signaling pathway, and cell adhesion molecules (CAMs) (Figure 3(c)).

### 3.4. Construction of the PPI Network and Identification of the Hub Genes

**Figure 4:**
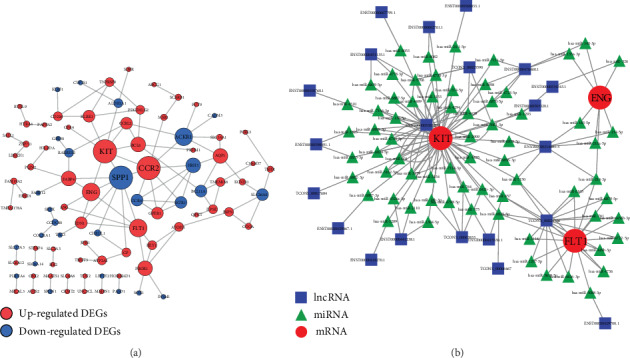
Construction of the lncRNA-miRNA-mRNA ceRNA network in placental tissues of EOPE and the control group. (a) PPI networks of the DEGs. Node size is proportional to the degree of the node itself. Edge width is proportional to the combined degree between genes. (b) lncRNA-miRNA-mRNA ceRNA network. Nodes with different colors and shapes represent different RNA types. lncRNA: long noncoding RNA; miRNA: microRNA; mRNA: messenger RNA; ceRNA: competing endogenous RNA; EOPE: early-onset preeclampsia; PPI: protein-protein interaction.

By using the online database STRING and the software Cytoscape, we constructed a PPI network of the DEGs. A total of 91 DEGs were mapped into this network (Figure 4(a)).

We evaluated their degree of connection and identified 6 hub genes including secreted phosphoprotein 1 (SPP1), C-C chemokine receptor type 2 (CCR2), receptor tyrosine kinase (KIT), endoglin (ENG), atypical chemokine receptor 1 (ACKR1), and fms related tyrosine kinase 1 (FLT1).

### 3.5. Construction of the ceRNA Network

To establish the ceRNA network, lncRNA-mRNA pairs which were positively correlated and contain the hub genes were obtained. Based on the results of the online prediction, we obtained the miRNAs that might interact with both DElncRNAs and hub genes. By integrating them, a ceRNA network containing 21 lncRNAs, 3 mRNAs, and 69 miRNAs was finally constructed (Figure 4(b)).

### 3.6. Validation of the DElncRNAs in the ceRNA Network by qRT-PCR in Placental Tissues

**Figure 5:**
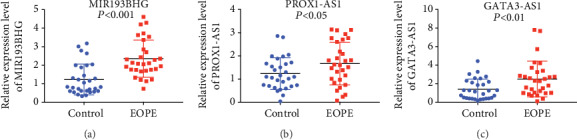
QRT-PCR validated three DElncRNAs in placental tissues of EOPE and the control group. The expression levels of (a) MIR193BHG, (b) PROX1-AS1, and (c) GATA3-AS1 were significantly elevated in placental tissues of EOPE (*n* = 30) compared with the control group (*n* = 30). EOPE: early-onset preeclampsia; DElncRNA: differentially expressed lncRNA. Data are presented as mean ± SD. *P* < 0.05 was considered statistically significant.

After a comprehensive analysis, 3 lncRNAs in the ceRNA network were validated in placental tissues from 30 EOPE patients and 30 normal controls. The clinical characteristics of these patients are presented in Table 2. We found that the relative expression levels of lncRNA MIR193BHG, PROX1-AS1, and GATA3-AS1 were significantly increased in EOPE compared with the normal controls (Figures 5(a), 5(b), and 5(c)). These results indicated that lncRNA MIR193BHG, PROX1-AS1, and GATA3-AS1 might act as ceRNAs to exert significant roles in regulating the expression of key genes in EOPE.

### 3.7. The Clinical Value of lncRNA MIR193BHG, PROX1-AS1, and GATA3-AS1 for Predicting the Risk of EOPE

**Figure 6:**
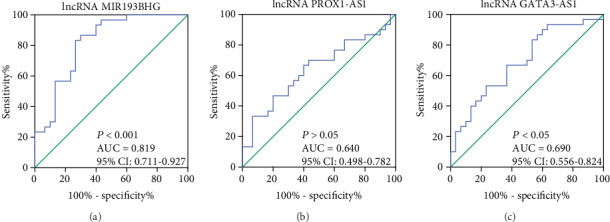
ROC curve analysis of the diagnostic value of the three validated lncRNAs. ROC curve analysis of (a) lncRNA MIR193BHG, (b) PROX1-AS1, and (c) GATA3-AS1 for diagnosis of EOPE. AUC: area under curve; EOPE: early-onset preeclampsia; CI: confidence interval. *P* < 0.05 was considered statistically significant.

To assess the clinical value of lncRNA MIR193BHG, PROX1-AS1, and GATA3-AS1 in the diagnosis of EOPE, ROC curves were performed. The area under the ROC curve (AUC) values of lncRNA MIR193BHG and lncRNA GATA3-AS1 for predicting the risk of EOPE were 0.819 (95% CI: 0.711–0.927, *P* < 0.001) and 0.690 (95% CI: 0.556–0.824, *P* < 0.05), respectively (Figures 6(a) and 6(c)). The AUC of lncRNA PROX1-AS1 was 0.640 (95% CI: 0.498-0.782, *P* > 0.05) as shown in Figure 6(b). These data suggested that lncRNA MIR193BHG and lncRNA GATA3-AS1 might be biomarkers for EOPE.

## 4. Discussion

lncRNAs have historically been regarded as junk DNA in the human genome [16]. However, increasing lncRNAs have been found to be involved in many important regulatory processes and may serve as biomarkers for various diseases. lncRNA can regulate the expression of target genes through a variety of mechanisms. The most widely studied is the ceRNA mechanism, which lncRNA uses to regulate the expression of downstream genes by competitively binding to miRNA through miRNA response elements [17].

In order to construct the ceRNA regulatory network of lncRNA-miRNA-mRNA, we first screened out the key genes (SPP1, CCR2, KIT, ENG, ACKR1, and FLT1) altered in placental tissues of EOPE based on the mRNA microarray data and online tool. FLT1 and ENG have been widely investigated in EOPE. FLT1, also known as vascular endothelial growth factor receptor 1 (VEGFR-1), binds to VEGF and is mainly involved in angiogenesis. Many researches revealed that FLT1 and its soluble form were increased in placenta or maternal circulation among women with EOPE [18]. Soluble FLT1 is the most promising placenta-derived predictive biomarker for EOPE [19]. High levels of soluble FLT1 in maternal circulation act as antiangiogenic factors and lead to endothelial dysfunction as well as end-organ dysfunction in PE [20]. ENG is another molecule involved in angiogenesis. The levels of soluble ENG in the maternal circulation are closely related to the severity of pregnancy-induced hypertension and its adverse outcomes [21]. Administration of both soluble FLT1 and ENG in pregnant rats produces a severe preeclampsia-like symptom with hypertension, proteinuria, glomerular endotheliosis, and thrombocytopenia [22]. SPP1, also known as osteopontin (OPN), is located in the placental syncytiotrophoblasts and in the cytoplasm of capillary endothelial cells [23]. It may serve as a marker for placental bed remodeling [24]. Decreased expression of OPN has been reported to be involved in the pathogenesis of preeclampsia [23–25]. CCR2 are involved in Th1 and Th2 immunity activation which might contribute to the development of preeclampsia [26]. KIT gene encodes the human homolog of the protooncogene C-kit and is also known as CD117. C-kit was found in placental tissues and in the maternofetal interface which suggested that it might be involved in the differentiation and development of placenta [27, 28]. ACKR1 plays a role in neutrophil migration and is expressed on endothelial cells. High levels of inflammatory cytokines such as IL-8 and TNF*α* in preeclampsia is correlated with the lack of Duffy expression which may contribute to increased neutrophil activation in the pathogenesis of preeclampsia [29].

Considering the crucial roles of these key genes in the pathogenesis of preeclampsia, it is of great significance to construct a lncRNA-related ceRNA network consisting of these genes. Based on the results of the online prediction, a new ceRNA network containing 21 lncRNAs, 3 mRNAs, and 69 miRNAs was finally constructed. Through this network, not only can we obtain important lncRNAs, miRNAs, and mRNAs related to the occurrence and progression of EOPE, but we can also further understand its underlying mechanism, which will contribute to the early diagnosis, treatment, and prognosis of EOPE. After qRT-PCR validation and ROC curve analysis, we found that MIR193BHG and GATA3-AS1 were elevated in the placental tissues of EOPE and had diagnostic value in distinguishing placental tissues of EOPE from the control group. Thus, these two lncRNAs could be worthy of further study when seeking novel biomarkers for predicting and diagnosing EOPE.

This study has its limitations. First, the function of lncRNA depends on its subcellular localization [17]. lncRNAs located in the cytoplasm may act as ceRNAs to regulate the expression of the target genes. But for the lncRNAs we screened, we did not verify their subcellular localization. Second, we only validated the expression of lncRNAs in placental tissues from 30 EOPE patients and 30 normal controls. This small sample number may reduce the reliability of our findings. Third, we only validated the expression of selected lncRNAs in placental tissues. Determining whether these lncRNAs participate in the pathogenesis of EOPE as ceRNAs and what are the specific mechanisms requires further experiments *in vitro* and *in vivo*.

## 5. Conclusion

In summary, we constructed a lncRNA-related ceRNA network that regulated the expressions of key genes in EOPE. Increased expression of lncRNA MIR193BHG and GATA3-AS1 in placental tissues had potential values to be biomarkers for diagnosing EOPE.

## Figures and Tables

**Table 1:** Clinical characteristics of EOPE and controls for microarray.

	Age (year)	BMI (kg/m^2^)	Week gestation (week)	Systolic BP (mmHg)	Diastolic BP (mmHg)	Proteinuria (g/24 h)	Birth weight (g)
C1	30	28.21	36 + 1	106	60	—	2100
C2	30	27.34	37 + 3	120	73	—	2700
C3	28	26.74	37 + 1	117	74	—	2550
C4	28	25.40	36 + 3	120	80	—	2400
P1	29	26.57	31 + 5	171	110	7.86	1300
P2	26	28.18	35 + 1	175	108	9.63	1800
P3	28	27.24	34 + 5	150	95	6.52	1500
P4	21	29.68	34 + 4	178	111	4.22	1680

C: control; P: early-onset preeclampsia; EOPE: early-onset preeclampsia; BMI: body mass index; BP: blood pressure.

**Table 2:** Clinical characteristics of EOPE and controls.

Clinical features	EOPE (*n* = 30)	Control (*n* = 30)	*P* value
Maternal age (years)	33.40 ± 5.24	34.17 ± 5.72	0.59
Gestational age at delivery (weeks)	34.17 ± 5.72	38.13 ± 0.44	<0.001^∗^
Maternal BMI (kg/m^2^)	33.38 ± 2.10	38.86 ± 0.47	0.36
Proteinuria (g/24 h)	4.61 ± 2.98	0	<0.001^∗^
Systolic BP (mmHg)	166.57 ± 15.78	113.70 ± 11.08	<0.001^∗^
Diastolic BP (mmHg)	98.37 ± 11.91	76.70 ± 9.50	<0.001^∗^
Neonatal birth weight (g)	1800.40 ± 526.64	3306.07 ± 238.38	<0.001^∗^

Data are presented as mean ± SD. ^∗^*P* < 0.05, *t*-test. EOPE: early-onset preeclampsia; BP: blood pressure; BMI: body mass index.

## Data Availability

The data used to support the findings of this study are available from the corresponding author upon request.
